# Supplementary material for: Hsp90 Inhibition: A Promising Therapeutic Approach for ARSACS
Source: Int J Mol Sci. 2021 Oct 29;22(21):11722. doi: 10.3390/ijms222111722 (PMC8584178; doi:10.3390/ijms222111722)
Supplement: Supplementary file 1 [file ijms-22-11722-s001.zip › ijms-1281637-supplementary.pdf]

Supplementary materials

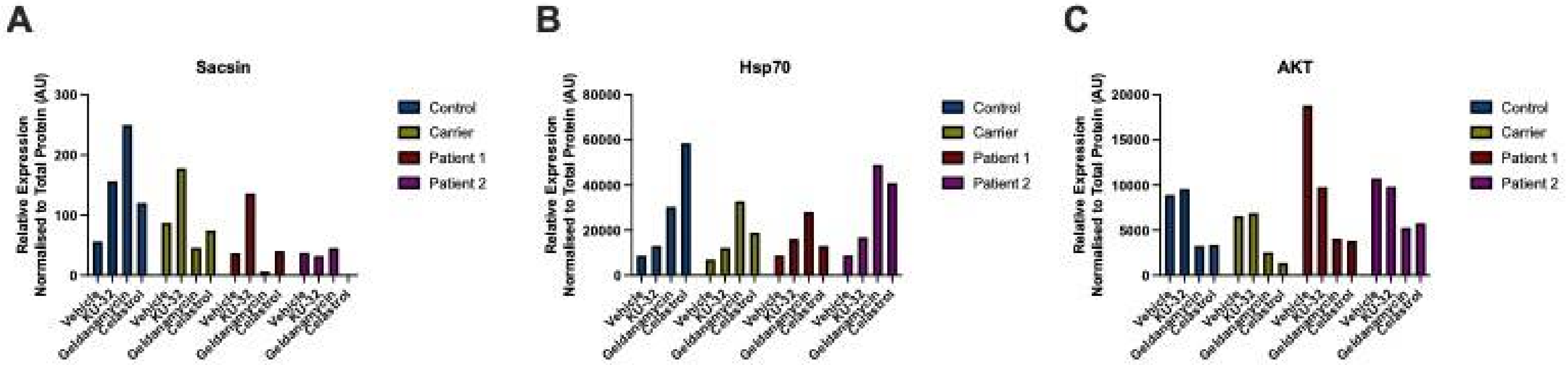

Figure S1. Immunoblot analysis of sacsin, Hsp70 and AKT expression in Control, Carrier and Patient fibroblast lines treated with Hsp90 inhibitors. Fibroblast lines were treated with DMSO vehicle, 100 nM KU-32, 500 nM geldanamycin or 1 μM celastrol for 24 h prior to cell lysis. KU-32 and celastrol are C-terminal Hsp90 inhibitors, whilst geldanamycin is an N-terminal Hsp90 inhibitor. **(A)** Densiometric quantitative analysis of sacsin expression shows a relative increase in sacsin expression across three out of four cell lines. **(B)** Hsp70 is a signature of the heat-shock response, and Hsp90 inhibition leads to an increase in Hsp70 levels. Densiometric quantitative analysis shows that KU-32 can induce Hsp70 expression at the concentration used in this study, but not as potently as geldanamycin or celastrol. **(C)** AKT is a client of Hsp90, and so its levels should be reduced upon Hsp90 inhibition. Geldanamycin and celastrol reduce AKT levels compared to Vehicle; however, there is limited degradation of AKT with KU-32 treatment, consistent with previous reports [1].

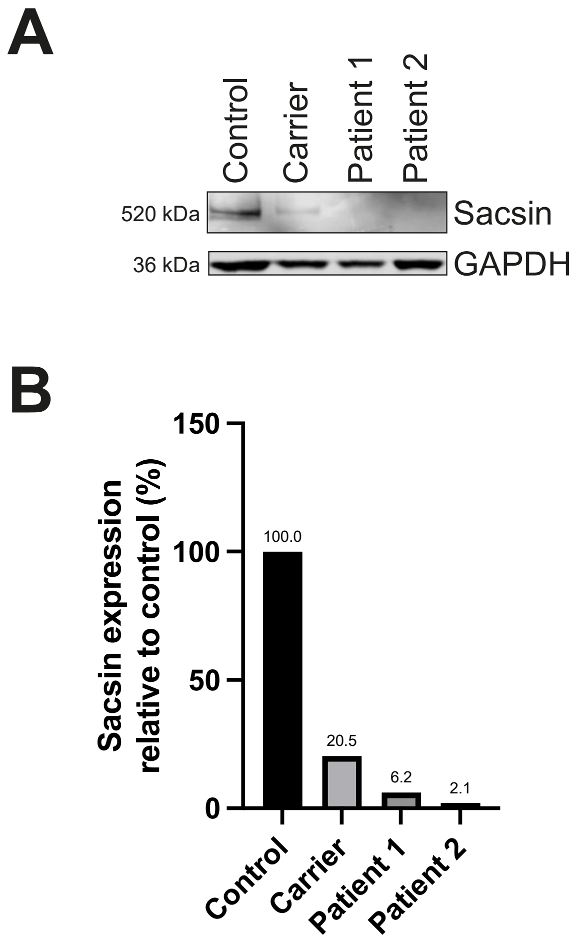

Figure S2. Sacsin expression in fibroblast lines used in this study. Immunoblot analysis of whole cell lysates from Control, Carrier, Patient 1 and Patient 2 cell lines. **(A)** Immunoblot probed with antibodies to sacsin and GAPDH. **(B)** Densiometric quantitative analysis of immunoblot showing sacsin expression relative to the band intensity of the Control Line. Loading was normalised using Total Protein Stain. Sacsin expression in Patient Lines 1 and 2 was severely reduced, whilst expression in the Carrier Line was intermediate.

References

1. Urban, M.J.; Li, C.; Yu, C.; Lu, Y.; Krise, J.M.; McIntosh, M.P.; Rajewski, R.A.; Blagg, B.S.J.; Dobrowsky, R.T. Inhibiting Heat-Shock Protein 90 Reverses Sensory Hypoalgesia in Diabetic Mice. *ASN Neuro* **2010**, *2*, e00040, doi:10.1042/AN20100015.
